# Supplementary material for: The ER membrane protein complex acts as a chaperone to promote the biogenesis of multi-bundle membrane proteins
Source: bioRxiv. 2026 Jan 15:2026.01.14.699575. Preprint. [Version 1] doi: 10.64898/2026.01.14.699575 (PMC12871136; doi:10.64898/2026.01.14.699575)
Supplement: 1 [file NIHPP2026.01.14.699575V1-supplement-1.pdf]

bioRxiv preprint doi: https://doi.org/10.64898/2026.01.14.699575; this version posted January 15, 2026. The copyright holder for this preprint (which was not certified by peer review) is the author/funder, who has granted bioRxiv a license to display the preprint in perpetuity. It is made available under aCC-BY 4.0 International license.

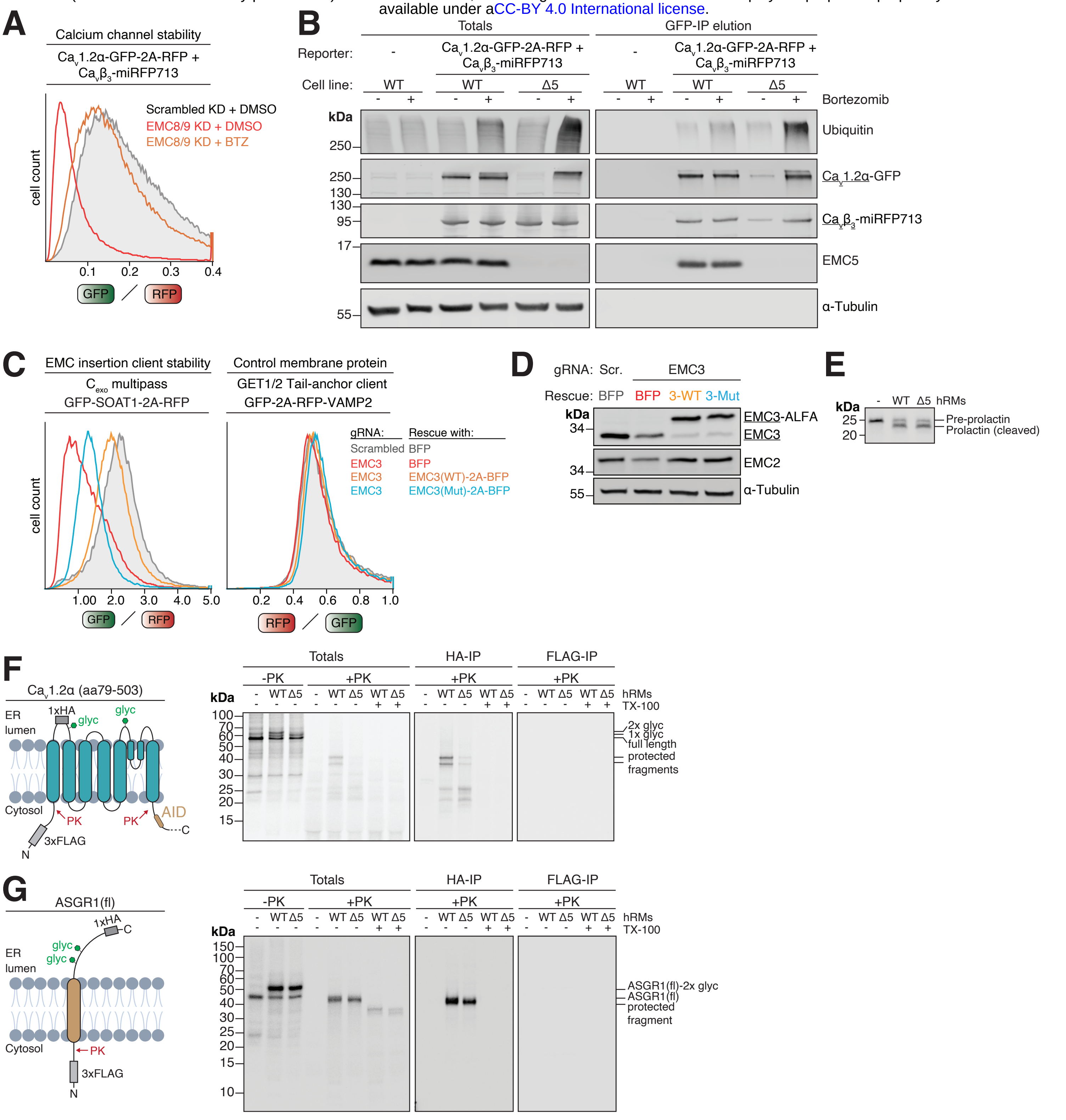

Figure S1. Characterization of Cav1.2α's EMC dependence.
A) 72h scrambled or EMC8+9 double siRNA knockdown in stable RPE1 $Ca_v1.2α•Ca_vβ_3$ reporter cells. Cells were additionally treated with 10 nM proteasome inhibitor bortezomib or DMSO solvent control for 16h before analysis of the GFP/RFP ratio by flow cytometry. **B)** HEK293 WT, or HEK293 WT and EMC5 KO (Δ5) cells stably expressing the $Ca_v1.2α•Ca_vβ_3$ reporter depicted in Fig. 1B were treated with DMSO or 10 nM bortezomib for 16h. Cells were harvested, lysed in detergent and subjected to anti-GFP nanobody purification of $Ca_v1.2α$-GFP. Total cell lysates and protease elution of the GFP-IP were analyzed by western blotting with the indicated antibodies. Note that $Ca_v1.2$ α-GFP is largely degraded in EMC5 KO cells, but can be stabilized by bortezomib treatment. The $Ca_v1.2α$ channels that accumulate are heavily ubiquitinated, indicating that $Ca_v1.2α$ is ubiquitinated and degraded in the absence of

bioRxiv preprint doi: https://doi.org/10.64898/2026.01.14.699575; this version posted January 15, 2026. The copyright holder for this preprint (which was not certified by peer review) is the author/funder, who has granted bioRxiv a license to display the preprint in perpetuity. It is made available under aCC-BY 4.0 International license.

Figure S1. continued

the EMC. **C)** Knockdown of EMC3 by CRISPRi in RPE1 dCas9-BFP-KRAB cells transiently transduced with C_{exo} multipass EMC insertase client reporter SOAT1 (Wu *et al.*, 2024) or EMC-independent tail-anchored membrane protein reporter VAMP2, a client of the GET1/2 insertase. Knockdown was rescued with either just BFP or BFP separated by a 2A site from ALFA-tagged wild-type (WT) EMC3 or the insertase-deficient EMC3 R31A+R180A mutant (Mut). The GFP/RFP (SOAT1) or RFP/GFP (VAMP2) ratios of BFP^{+} cells were determined by flow cytometry and are depicted as histograms. **D)** Experiment as in C, but analysis of total cell lysates by western blotting with the indicated antibodies. **E)** WT and EMC5 KO (Δ5) hRMs are equally active in Sec61-dependent protein translocation. ^{35}S-methionine-labled bovine preprolactin carrying a cleavable signal sequence was translated in rabbit reticulocyte lysate in the presence or absence of human rough ER membranes (hRMs) prepared from either wild-type or Δ5 HEK 293 cells. **F)** Insertion defect of Ca_v1.2α in EMC5 KO (Δ5) membranes. As in Fig. 1F, but with Ca_v1.2α (amino acids [aa] 79-537) carrying both an N-terminal 3xFLAG tag, as well as a 1x HA tag inserted into the lumenal loop between TM1 and TM2. The latter makes an otherwise occluded (Fig. 1F) glycosylation (glyc) site accessible. Non-incorporated as well as cytosolically accessible protein portions were digested with proteinase K (PK) in the presence or absence of Triton-X-100 (TX-100) to solubilize the hRM membrane. The resulting protease protected fragments (PFs) were subjected to denaturing anti-HA and anti-FLAG immunoprecipitations (IP). **G)** No insertion defect of ASGR1, a Sec61-dependent single-pass type II membrane protein, was observed in Δ5 hRMs. Assay as in F, but with full length (fl) ASGR1 carrying an N-terminal 3xFLAG and C-terminal 1xHA tag.

bioRxiv preprint doi: https://doi.org/10.64898/2026.01.14.699575; this version posted January 15, 2026. The copyright holder for this preprint (which was not certified by peer review) is the author/funder, who has granted bioRxiv a license to display the preprint in perpetuity. It is made available under aCC-BY 4.0 International license.

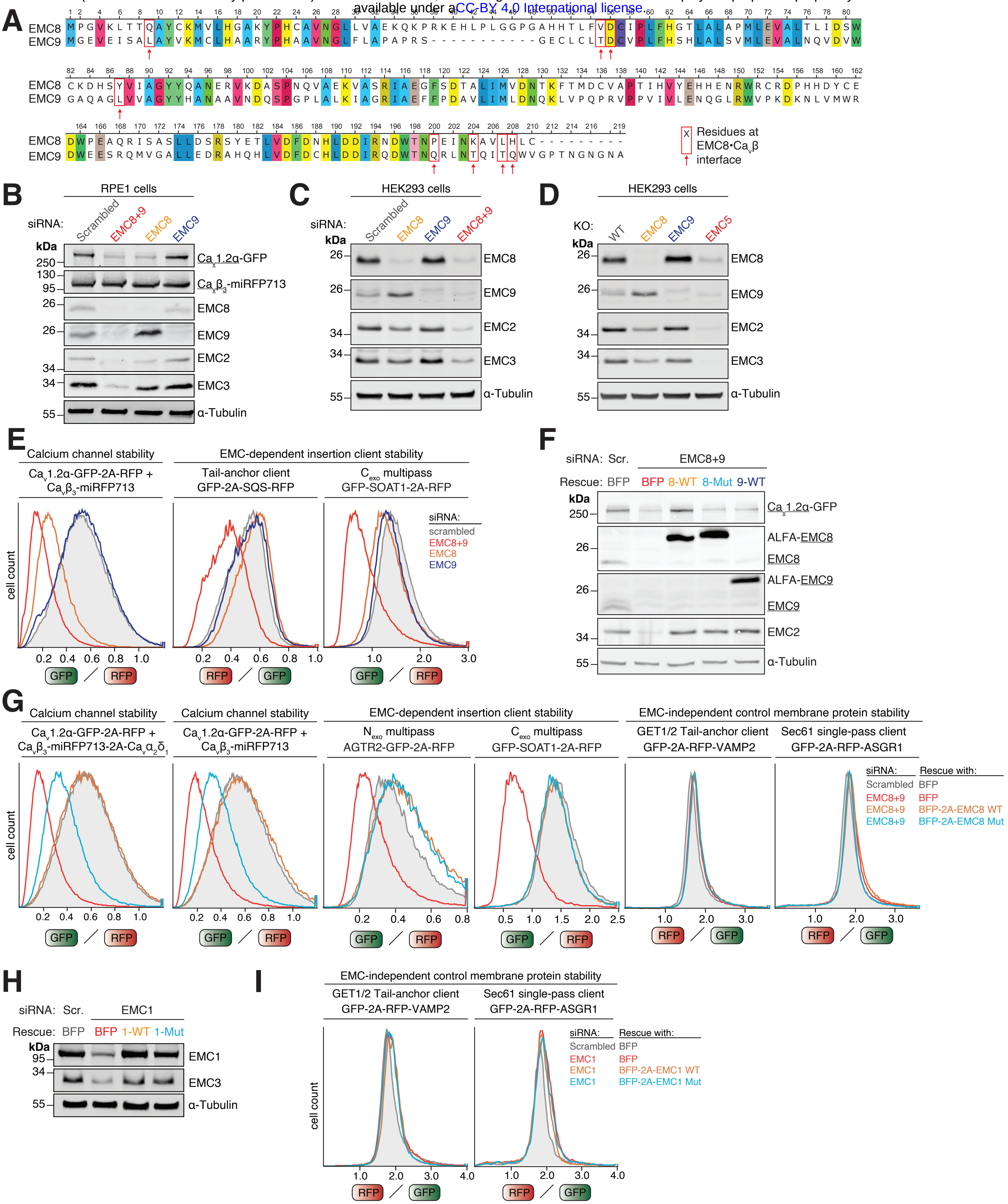

bioRxiv preprint doi: https://doi.org/10.64898/2026.01.14.699575; this version posted January 15, 2026. The copyright holder for this preprint (which was not certified by peer review) is the author/funder, who has granted bioRxiv a license to display the preprint in perpetuity. It is made available under aCC-BY 4.0 International license.

Figure S2. Characterization of EMC's chaperone interfaces.

A) Sequence alignment of human EMC8 and EMC9 generated with Clustal Omega (Madeira *et al.*, 2024) and visualized with Unipro UGENE (Okonechnikov *et al.*, 2012). Both paralogs display ~40% sequence identity. Identical positions are highlighted. EMC8 residues that contact $Ca_v\beta_3$ in the EMC•Ca_v1.2α•$Ca_v\beta_3$ co-structure (PDB 8EOI, Chen *et al.*, 2023) are highlighted in red boxes to illustrate that 7/8 of these residues are altered in EMC9. **B)** 72h scrambled, EMC8, EMC9 or EMC8+9 double siRNA knockdown in RPE1 cells. Total cell lysates were analyzed by western blotting with the indicated antibodies. **C)** As in B, but in HEK 293T cells. **D)** Western blot analysis of total cell lysates prepared from HEK 293T WT, EMC8 KO, EMC9 KO or EMC5 KO cells. **E)** 72h scrambled, EMC8, EMC9 or EMC8+9 double siRNA knockdown in stable RPE1 Ca_v1.2α•$Ca_v\beta_3$ reporter cells or RPE1 cells transiently transduced with EMC-dependent insertase client reporters SQS and SOAT1. The GFP/RFP (Ca_v1.2α, SOAT1) or RFP/GFP (SQS) ratios of the resulting cells were determined by flow cytometry and are depicted as histograms. **F)** 72h scrambled or EMC8+9 double siRNA knockdown in stable RPE1 Ca_v1.2α•$Ca_v\beta_3$ reporter cells. 24h post siRNA transfection, cells were transduced with lenti-viral rescue constructs encoding either just BFP or BFP separated by a 2A site from ALFA-tagged EMC8 WT, EMC8 K204A, L207A, H208A mutant (Mut) or EMC9 WT. Total cell lysates were analyzed by western blotting with the indicated antibodies. Note that all variants rescue EMC assembly as judged by the levels of EMC8's partner subunit EMC2. **G)** As in F but in stable RPE1 Ca_v1.2α•$Ca_v\beta_3$ reporter cells, Ca_v1.2α•$Ca_v\beta_3$•$Ca_v\alpha_2\delta_1$ reporter cells or RPE1 cells transiently transduced with the indicated fluorescent reporters. The GFP/RFP or RFP/GFP ratios of BFP^+ cells were determined by flow cytometry and are depicted as histograms. **H)** 72h scrambled or EMC1 siRNA knockdown in stable RPE1 Ca_v1.2α•$Ca_v\beta_3$ reporter cells. 24h after siRNA transfection, cells were transduced with lenti-viral rescue constructs encoding either just BFP or BFP separated by a 2A site from the prolactin signal sequence (Prl(ss))-ALFA-tagged EMC1 WT (23-end) or EMC1 D961A, R981L mutant (Mut) (23-end). Total cell lysates were analyzed by western blotting with the indicated antibodies. Note that EMC1(Mut) rescues EMC assembly as judged by the levels of EMC core subunit EMC3. **I)** As in H but in RPE1 cells transiently transduced with the indicated fluorescent reporters. The RFP/GFP ratios of the BFP^+ cells were determined by flow cytometry and are depicted as histograms.

bioRxiv preprint doi: https://doi.org/10.64898/2026.01.14.699575; this version posted January 15, 2026. The copyright holder for this preprint (which was not certified by peer review) is the author/funder, who has granted bioRxiv a license to display the preprint in perpetuity. It is made available under aCC-BY 4.0 International license.

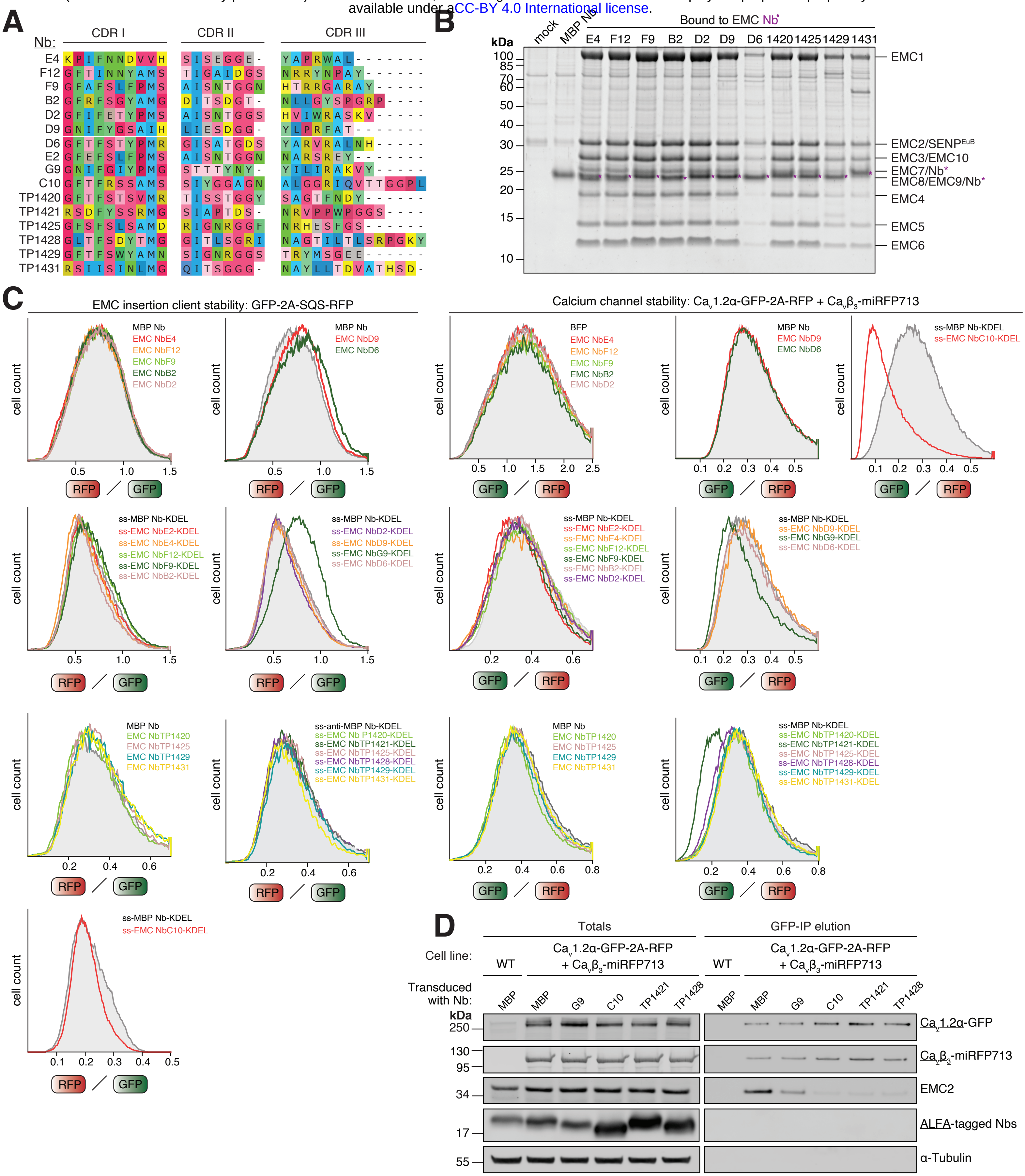

bioRxiv preprint doi: https://doi.org/10.64898/2026.01.14.699575; this version posted January 15, 2026. The copyright holder for this preprint (which was not certified by peer review) is the author/funder, who has granted bioRxiv a license to display the preprint in perpetuity. It is made available under aCC-BY 4.0 International license.

Figure S3. Characterization of the anti-EMC nanobody toolbox.

A) Focused sequence alignment of the 16 different anti-EMC nanobody (Nb) classes characterized in this study. Only the sequence stretches encompassing the complementarity-determining regions (CDRs) I-III, which constitute the antigen-binding loops of these Nbs, are depicted. **B)** ALFA-GFP-SUMO^{Eu}-tagged anti-MBP control or anti-EMC Nbs were immobilized via a biotinylated, SUMOstar protease-cleavable anti-ALFA tag Nb on Streptavidin magnetic beads. The beads were incubated with a GDN-solubilized Expi293 total cell lysate. After washing, proteins bound to the control or anti-EMC Nbs were specifically eluted by native SENP^{EuB} cleavage. The eluate was analyzed by SDS-PAGE and SYPRO Ruby staining. Note that all anti-EMC Nbs specifically purified the nine EMC subunits. Nbs D6, TP1429 and TP1431 had lower affinity and isolated less EMC. **C)** Flow cytometry assay in stable RPE1 $Ca_v1.2\alpha \bullet Ca_v\beta_3$ reporter cells or RPE1 cells transiently transduced with EMC-dependent insertase client reporter SQS. These cell lines were transduced to express the indicated Nbs either from a BFP-2A-Nb cassette in the cytosol or from a BFP-2A-Prl(ss)-Nb-KDEL cassette in the ER lumen. The GFP/RFP or RFP/GFP ratios of BFP$^+$ cells are depicted as histograms. Note, that inhibitory Nbs G9, C10, TP1421 and TP1428 also inhibited chaperone function when targeted to the ER lumen. We believe this occurs because small, fast-folding proteins like Nbs might occasionally escape translocation into the ER lumen and accumulate at sufficient levels in the cytosol to cause the observed inhibitory effect. **D)** Stable RPE1 $Ca_v1.2\alpha \bullet Ca_v\beta_3$ reporter cells were transduced to express the indicated anti-MBP control or inhibitory anti-EMC Nbs in the cytosol. Cells were lysed in detergent and subjected to anti-GFP Nb purification of $Ca_v1.2\alpha$-GFP to assess EMC co-purification in the presence of these Nbs. Total cell lysates and protease elution of the GFP-IP were analyzed by western blotting with the indicated antibodies.

bioRxiv preprint doi: https://doi.org/10.64898/2026.01.14.699575; this version posted January 15, 2026. The copyright holder for this preprint (which was not certified by peer review) is the author/funder, who has granted bioRxiv a license to display the preprint in perpetuity. It is made available under aCC-BY 4.0 International license.

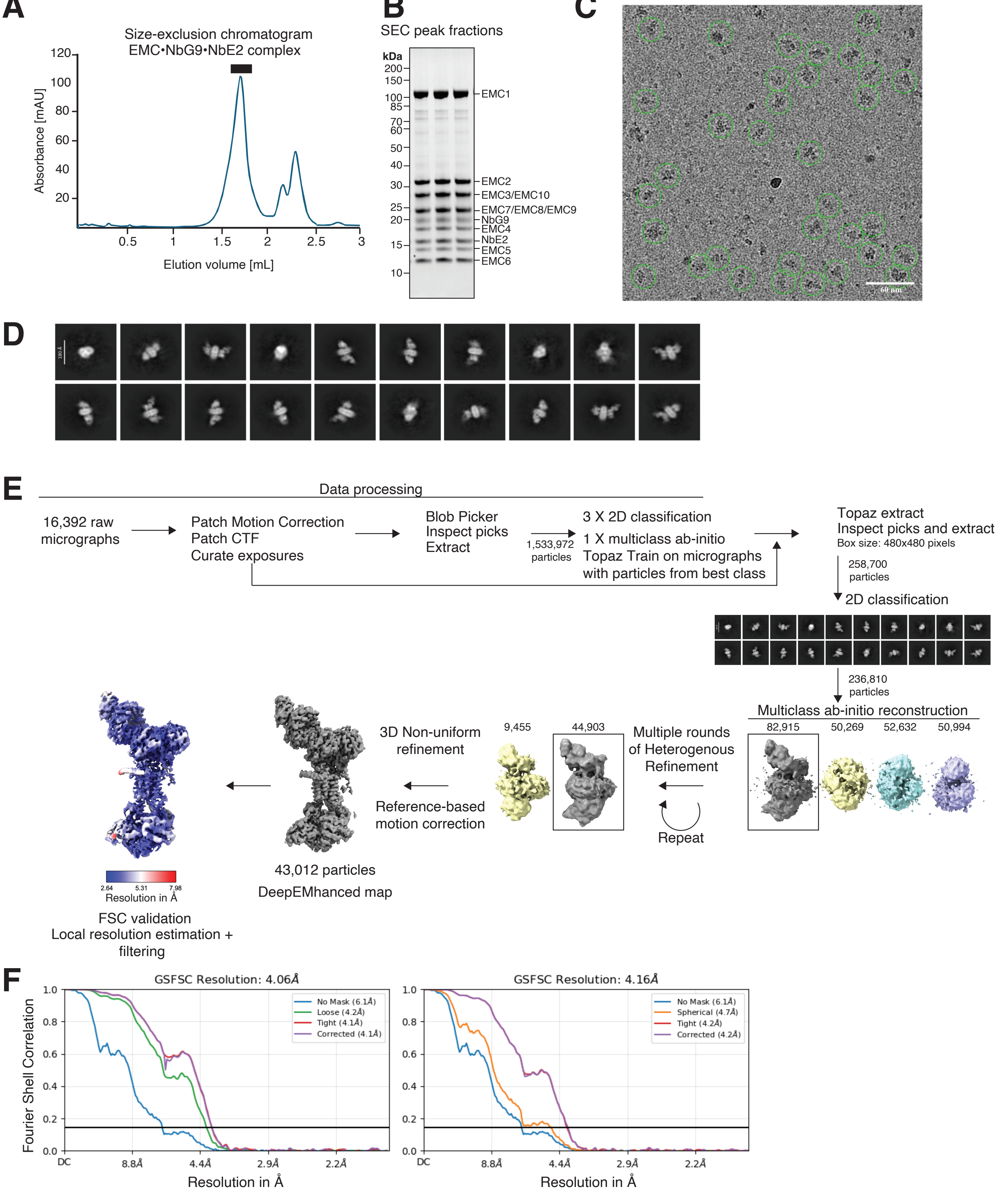

bioRxiv preprint doi: https://doi.org/10.64898/2026.01.14.699575; this version posted January 15, 2026. The copyright holder for this preprint (which was not certified by peer review) is the author/funder, who has granted bioRxiv a license to display the preprint in perpetuity. It is made available under aCC-BY 4.0 International license.

Figure S4. Cryo-EM structure of the human EMC bound to two nanobodies.

A) Endogenous human EMC was purified from Expi293 cell lysate using biotinylated, $SENP^{EuB}$-cleavable anti-EMC NbE2 and then mixed with purified inhibitory anti-EMC NbG9. The resulting complex was further purified by size-exclusion chromatography (SEC) on a Superose 6 Increase 3.2/300 column. **B)** Analysis of the SEC peak fractions from A by SDS-PAGE and SYPRO Ruby staining. **C)** Representative cryoEM micrograph collected using Titan Krios G2 operating at 300KeV on Falcon 4i detector and SelctrisX energy filter. Particles representing the EMC•NbG9•NbE2 complex picked using manual picker in cryoSPARC are highlighted. **D)** Representative 2D class averages generated during data processing. **E)** General cryoEM data processing workflow employed for obtaining the structure of the EMC in complex with Nbs G9 and E2. The local resolution map was calculated using cryoSPARC local resolution estimation and local resolution filtering methods and ranges from 2.6 Å to 7.9 Å resolution. **F)** Fourier Shell Correlation (FSC) of the final 4.06 Å cryo-EM map of the EMC•NbG9•NbE2 complex with different masks from the Non-uniform refinement and FSC validation, respectively.

bioRxiv preprint doi: https://doi.org/10.64898/2026.01.14.699575; this version posted January 15, 2026. The copyright holder for this preprint (which was not certified by peer review) is the author/funder, who has granted bioRxiv a license to display the preprint in perpetuity. It is made available under aCC-BY 4.0 International license.

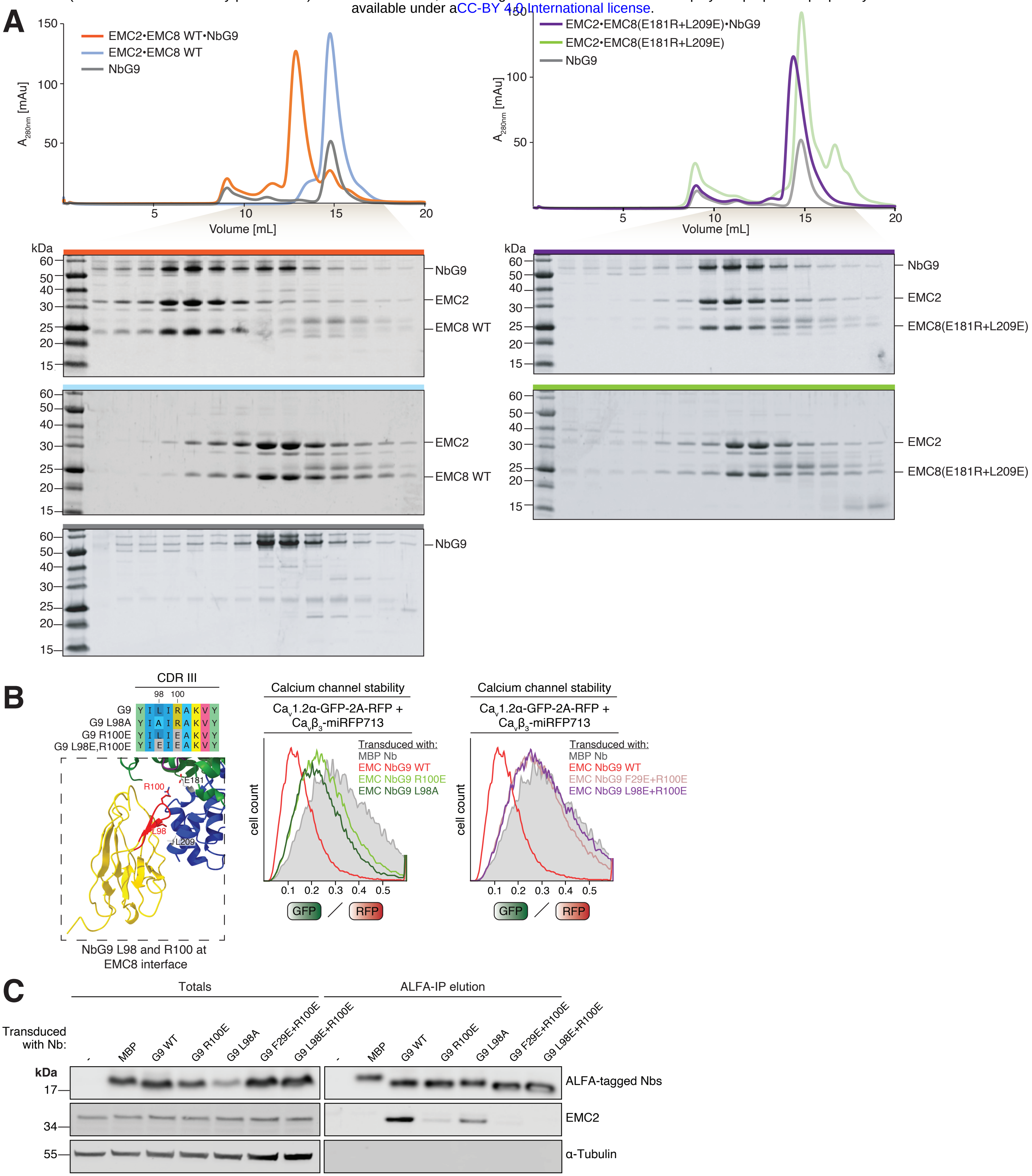

bioRxiv preprint doi: https://doi.org/10.64898/2026.01.14.699575; this version posted January 15, 2026. The copyright holder for this preprint (which was not certified by peer review) is the author/funder, who has granted bioRxiv a license to display the preprint in perpetuity. It is made available under aCC-BY 4.0 International license.

Figure S5. Validation of the EMC•NbG9 cryo-EM structure.

A) (*Left*) Superdex 200 10/300 size exclusion chromatograms of purified GFP-SUMO^{Eu}-tagged NbG9 (grey), purified EMC2•EMC8 complex (blue) or EMC2•EMC8•NbG9 complex (orange) are shown. Peak fractions were analyzed by SDS-PAGE and SYPRO Ruby staining. Gels are color-coded to match their respective chromatography runs. Note that NbG9 binds to the purified EMC2•EMC8 complex and results in a left shift to earlier elution volume, consistent with complex formation. (*Right*) As in A, but with an EMC8 variant that contains two mutations at the EMC8•Nb G9 interface. These mutations abolish ternary complex formation, validating the modeled NbG9 interface on the EMC in our cryo-EM structure. **B)** (*Left*) View of the NbG9•EMC8 interface in our cryo-EM model, highlighting CDR III in red and depicting residues L98 and R100 as sticks. The sequence of NbG9's CDR III and the mutations made below are highlighted on top. EMC8 residues E181 and L209 mutated in A) are highlighted as light grey sticks. (*Right*) Stable RPE1 $Ca_v1.2\alpha$•$Ca_v\beta_3$ reporter cells were transduced to express either anti-MBP control Nb, wild-type NbG9 or the indicated single and double mutants of NbG9 from a BFP-2A-Nb cassette in the cytosol. The GFP/RFP ratios of BFP$^+$ cells are depicted as histograms. F29 is located in CDR I. Note that mutations of NbG9 CDR residues abolish its inhibitory effect. **C)** RPE1 cells transduced with the indicated, ALFA-tagged Nbs were lysed in detergent and subjected to anti-ALFA tag Nb purification. Total cell lysates and ALFA Nb protease eluates were analyzed by western blotting with the indicated antibodies. Note that disruption of NbG9's CDR residues reduces its ability to interact with the EMC.

bioRxiv preprint doi: https://doi.org/10.64898/2026.01.14.699575; this version posted January 15, 2026. The copyright holder for this preprint (which was not certified by peer review) is the author/funder, who has granted bioRxiv a license to display the preprint in perpetuity. It is made available under aCC-BY 4.0 International license.

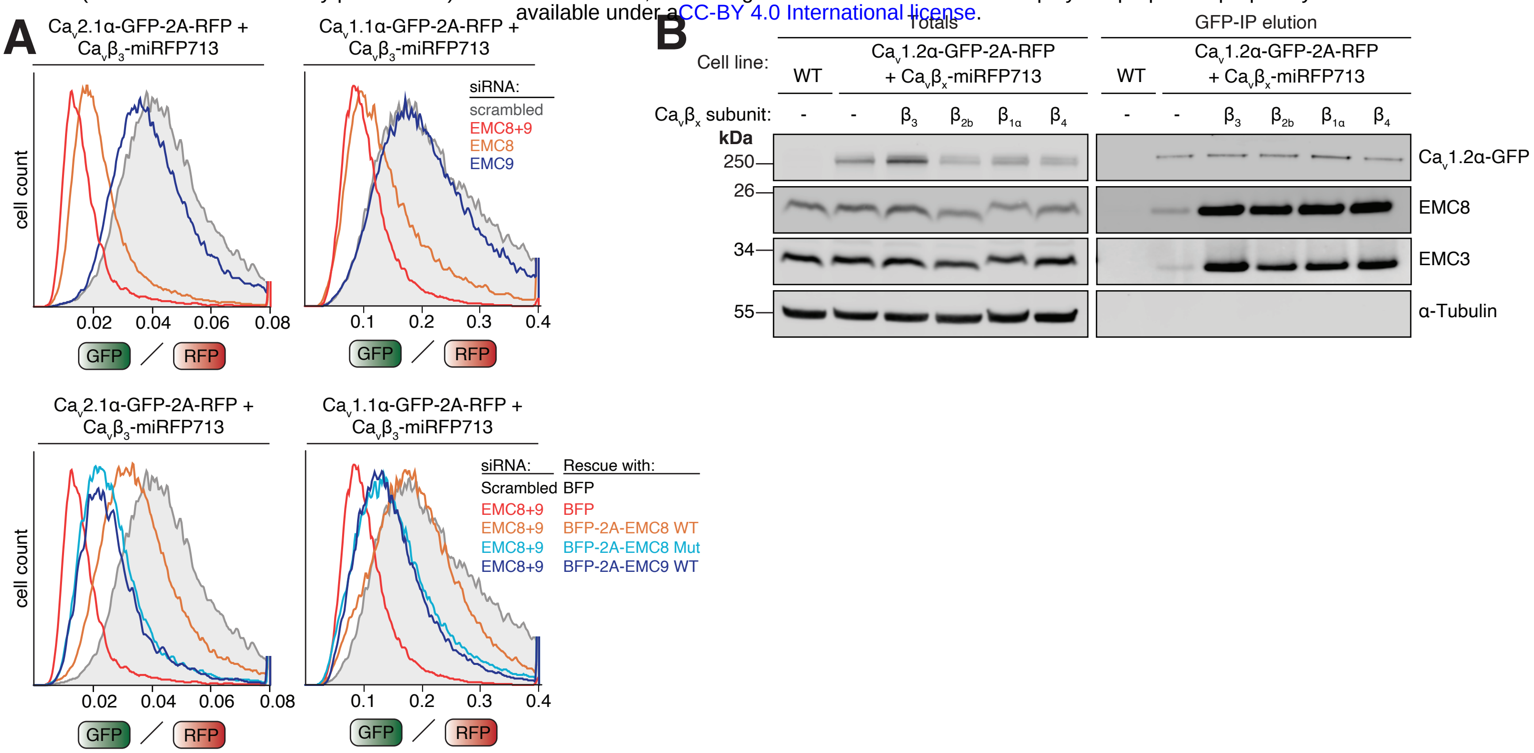

Figure S6. Biogenesis of brain and skeletal muscle calcium channels also relies on EMC chaperone function.

A) Experiments in stable RPE1 cells expressing the brain $Ca_v2.1\alpha \cdot Ca_v\beta_3$ channel (*left*) or skeletal muscle $Ca_v1.1\alpha \cdot Ca_v\beta_3$ channel reporter (*right*). (*Top*) 72h scrambled, EMC8, EMC9 or EMC8+9 double siRNA knockdown. The GFP/RFP ratios of the resulting cells were determined by flow cytometry and are depicted as histograms. (*Bottom*) 72h scrambled or EMC8+9 double siRNA knockdown. 24h after siRNA transfection, cells were transduced with lenti-viral rescue constructs encoding either just BFP or BFP separated by a 2A site from either EMC8 WT, EMC8 K204A, L207A, H208A mutant (Mut) or EMC9 WT. The GFP/RFP ratios of BFP^+ cells were determined by flow cytometry and are depicted as histograms. **B)** Stable RPE1 $Ca_v1.2\alpha$ reporter cells expressing the indicated $Ca_v\beta$ subunits fused to miRFP713 were lysed in detergent and subjected to anti-GFP Nb purification of $Ca_v1.2\alpha$-GFP to assess EMC co-purification in the presence of the different $Ca_v\beta$ subunits. Total cell lysates and protease elution of the GFP-IP were analyzed by western blotting with the indicated antibodies.

bioRxiv preprint doi: https://doi.org/10.64898/2026.01.14.699575; this version posted January 15, 2026. The copyright holder for this preprint (which was not certified by peer review) is the author/funder, who has granted bioRxiv a license to display the preprint in perpetuity. It is made available under aCC-BY 4.0 International license.

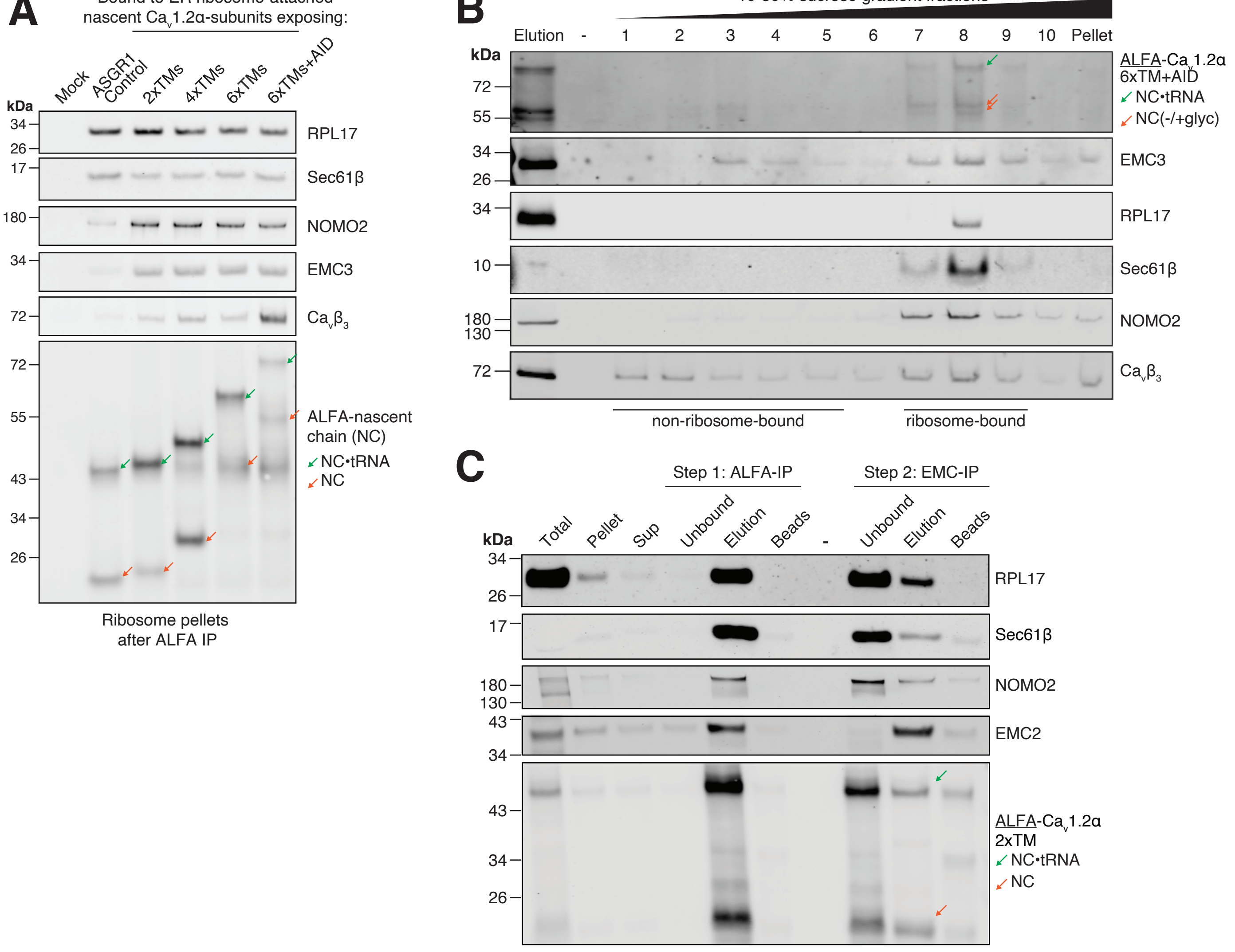

Figure S7. The EMC associates co-translationally with Ca_v1.2α-synthesizing ribosome•MPT complexes.
A) Assay as in Fig. 5C. Normalized ribosome pellets of ribosome-nascent chain complexes (RNCs) stalled while translating either an EMC-independent single-pass control protein ASGR1, or Ca_v1.2α exposing 2x, 4x, 6x or 6xTMs plus the AID-containing cytosolic linker between bundles I and II outside of the ribosome exit tunnel, were analyzed by western blotting with the indicated antibodies. A mock translation without RNA served as a background control. Note that EMC engagement with TM bundle I intermediates of Ca_v1.2α is constant. **B)** Stalled RNCs containing nascent Ca_v1.2α exposing 6xTMs+AID were purified by ALFA nanobody IP as described in Fig. 5B and the eluate was then overlayed onto a 10-50% sucrose gradient. After the run, eleven fractions were collected from the top of the gradient for analysis by western blotting. Note that ribosomes and ribosome-bound proteins peak around fraction 8. Both EMC and $Ca_v\beta_3$ were found to migrate with ribosomes in the sucrose gradient. **C)** Stalled RNCs containing Ca_v1.2α exposing 2xTMs were generated as described in Fig. 5B. After purification of ALFA-tagged RNCs using a SUMOstar-cleavable anti-ALFA-nanobody, the resulting protease eluate was subjected to a second IP with inert, $SENP^{EuB}$-cleavable anti-EMC NbE2. Samples of this 2-step IP were analyzed by western blotting with the indicated antibodies. Note that the EMC is bound to intact RNCs containing tRNA-associated nascent chain (NC), ribosomes (RPL17), the translocon (Sec61β) and MPT BOS complex subunit NOMO2.

bioRxiv preprint doi: https://doi.org/10.64898/2026.01.14.699575; this version posted January 15, 2026. The copyright holder for this preprint (which was not certified by peer review) is the author/funder, who has granted bioRxiv a license to display the preprint in perpetuity. It is made available under aCC-BY 4.0 International license.

Table S1. Protein sequences of anti-EMC nanobodies generated in this study.

Nanobody #	Sequence	Plasmid #	Addgene ID
E4	QVQLVESGGGLVQAGGSLRLSCAASKPIFNNDVVHWYRQAPGNQHDFVASISEGGETDYADFVKGRFSISRDNVGARVYLQMNNLKPEDTAVYYCYAPRWALWGQGTQVTVSS	pTP1321	TBD
F12	QVQLVESGGGLVQTGGSLRLSCAASGFTINNYAMSWYRQAPGKERELVATIGAIDGSTNYLDSVKGRFAISRDNAKNTVYLQMNSLKPEDTAVYYCNRRYNPAYWGQGTRVTVSS	pTP1322	TBD
F9	QVQLVESGGGLVQPGGSLRLSCAASGFAFSLFPMSWHRQAPGKERELVAAISNTGGNTNYADSVKGRFTISRDNAKNTAYLQMNSLKLEDTAVYYCHTRRGARAYWGQGTQVTVSS	pTP1329	TBD
B2	QVQLVESGGGVVQAGGSLRLSCAVSGFRFSGYAMGWYRQVPGKQRELVADITSDGTTNYADSVKGRFTISRDNARKTVYLQMSSLKPEDTAVYYCNLLGYSPGRPWGPGTQVTVSS	pTP1330	TBD
D2	QVQLVESGGGLVQPGGSLRLSCTASGFIFETYPMSWHRQAPGKERELVAAISNTGGSINYADSVKGRFTISRDNAKNTMYLQMNSLKPEDTAMYYCHVIWRASKVWGQGTRVTVSS	pTP1331	TBD
D9	QVQLVESGGGSVQAGGSLTLHCTTSGNIFYGSAIHWHRQVAGNTREWVALIESDGGTSYAGSVQGRFVVSRDTANTIVTLQMNSLKPEDTGTYYCYLPRFATWGQGTQVTVSS	pTP1374	TBD
D6	QVQLVESGGGLVQPGGSLRLSCAVSGFTFSTYPMRWYRQAPGKEREFVAGISATGDSSTYTHFAEERFIISRDNGKNTMYLQMNSLRPEDTAIYFCYARVRALNHWGQGTLVTVSS	pTP1378	TBD
E2	QVQLVESGGGLVQVGGSLRLSCAASGFEFSLFPMSWHRQAPGKEREFVAAISNTGGNTNYADSVKGRFTISRDNAKNTAYLQMNSLKPEDTAVYYCNARSREYWGQGTQVTVSS	pTP1320	TBD
G9	QVQLVESGGGLVQPGGSLRLSCAASGNIFGIYPMGWHRQAPGKQREFVASTTTYNYTSYADSAKGRFTIARDNAKNTVYLQMNSLKPEDTAIYYCYILIRAKVYRGQGTLVTVSS	pTP1376	TBD
C10	QVQLVESGGGLVQPGESLRLSCATSGFTFRSSAMSWVRQAPGKELEWVSSIYGGAGNTYYADAVKGRFTISRDNSENTLYLQMNSLLPEDTAVYYCALGGRIQVTTGGPLRGQGTQVTVSS	pTP1377	TBD
TP1420	QVQLVESGGGLVQPGGSLRLSCVASGFTFSTSVMRWHRQALGKERKLVATISSTGGYTYYVDSVKGRFTISRDNAKNTAYLQMNSLKPEDTAVYYCSAGTFNDYWGQGTQVTVSS	pTP1420	TBD
TP1421	QVQLVESGGGLVQAGESLGLSCVASRSDFYSSRMGWYRQAPGKQREWVASIAPTDSTYYEDSVKGRFTISRDNSKNTVYLRMNSLKPEDTAVYYCNRVPPWPGGSWGQGTQVTVSS	pTP1421	TBD
TP1425	QVQLVESGGGLAQPGGSLTLSCAASGFSFSLSAMDWYRQAPGKERELVARIGNRGGFKDYADSVKGRFTISRDNTRNTVYLQMNSLKPDDTAVYYCNRHESFGSWGQGTQVTVSS	pTP1425	TBD
TP1428	QVQLVESGGGLVQTGGSLILSCAASGLTFSDYTMGWFRQAPGKEREFVAGITLSGRITYYADSVKGRFTISRDNAKNMVYLQMNSLKPEDTALYYCNAGTILTLSRPGKYWGQGTQVTVSS	pTP1428	TBD
TP1429	QVQLVESGGGLVQPGGSLRLSCAASGFTFSWYAMNWVRQAPGKGLEWVSSIGNRGGSTSYADSVKGRFTISRDNAKNTLYLQMNSLKPEDTAVYYCTRYMSGEERGPGTQVTVSS	pTP1429	TBD
TP1431	QVQLVESGGGLVQAGGSLRLSCAASRSIISINLMGWYRQAPGKQREFVAQITSGGGTNYAESVKGRFTISRDSAKNTVNLQMNSLKPEDTAVYYCNAYLLTDVATHSDWGQGTQVTVSS	pTP1431	TBD

bioRxiv preprint doi: https://doi.org/10.64898/2026.01.14.699575; this version posted January 15, 2026. The copyright holder for this preprint (which was not certified by peer review) is the author/funder, who has granted bioRxiv a license to display the preprint in perpetuity. It is made available under aCC-BY 4.0 International license.

Table S2. Cryo-EM data collection, refinement, and validation statistics

	Consensus (EMDB-74981)
**Data collection and processing**	
Microscope	FEI Titan Krios
Voltage (kV)	300
Camera	Falcon 4i with Selectris X
Magnification	130,000
Defocus range (µm)	-0.6 to -2.0
Pixel size (Å/pix)	0.92
Electron exposure (e–/Å^2)	54.23
Number of frames per movie	66
Dose Rate (e-/pix/s)	8.93
Automation software	EPU
Number of micrographs	16,376
Initial particle images (no.)	1,533,972
Final particle images (no.)	43,012
Local resolution range (Å)	2.6 – 7.9
Map resolution range (Å, FSC=0.143)	4.16
**Refinement**	
Software (phenix.real_space_refine)	PHENIX 1.21.2-5419
Initial model used (PDB code)	8s9s+Alpha fold
Correlation coefficient (CC_{mask})	0.80
Map sharpening *B* factor (Å^2)	-76.7
Model composition	
Non-hydrogen atoms	20560
Protein residues	2585
Ligands	7 NAG
B factors (Å^2)	
Protein	391.48
Ligand	193.39
R.M.S deviations	
Bond lengths (Å) (# > 4σ)	0.002
Bond angles (°) (# > 4σ)	0.552
**Validation**	
MolProbity score	1.65
Clashscore	7.34
Poor rotamers (%)	0.00
Cß deviations (%)	0.00
CaBLAM outliers (%)	1.70
Ramachandran plot	
Favored (%)	96.28
Allowed (%)	3.72
Outliers (%)	0.00

bioRxiv preprint doi: https://doi.org/10.64898/2026.01.14.699575; this version posted January 15, 2026. The copyright holder for this preprint (which was not certified by peer review) is the author/funder, who has granted bioRxiv a license to display the preprint in perpetuity. It is made available under aCC-BY 4.0 International license.

Additional supplemental items:

Supplemental Video S1. Live cell imaging of calcium dynamics in cardiomyocytes transduced with control anti-MBP nanobody. Cytosolic BFP is expressed from the same lenti-viral vector as the nanobody and served as a transduction marker. GCaMP6f fluorescence in the green channel indicates calcium dynamics accompanying cardiomyocyte contraction.

Supplemental Video S2. Live cell imaging of calcium dynamics in cardiomyocytes transduced with control anti-EMC nanobody G9. Cytosolic BFP is expressed from the same lenti-viral vector as the nanobody and served as a transduction marker. GCaMP6f fluorescence in the green channel indicates calcium dynamics accompanying cardiomyocyte contraction.

Supplemental Video S3. Live cell imaging of calcium dynamics in cardiomyocytes transduced with control anti-EMC nanobody C10. Cytosolic BFP is expressed from the same lenti-viral vector as the nanobody and served as a transduction marker. GCaMP6f fluorescence in the green channel indicates calcium dynamics accompanying cardiomyocyte contraction.
